# Microbiota in cancer chemoradiotherapy resistance

**DOI:** 10.1002/ctm2.250

**Published:** 2020-12-31

**Authors:** Yan He, Qin Wen Liu, Hua Xin Liao, Wen Wen Xu

**Affiliations:** ^1^ MOE Key Laboratory of Tumor Molecular Biology and Guangdong Provincial Key Laboratory of Bioengineering Medicine National Engineering Research Center of Genetic Medicine Institute of Biomedicine College of Life Science and Technology Jinan University Guangzhou China

Dear Editor,

Cancer is a global challenge that threatens human health. Low treatment efficacy and resistance to chemoradiotherapy limits the treatment outcomes in cancer patients. The microbiota in the human body has a symbiotic relationship with the human body and plays an important role in cancer treatment. These microorganisms attach themselves to the mucosal surface of almost every organ, although most of them reside in the intestinal tract and oral cavity and are called gut microbiota and oral microbiota, respectively. Others live in different regions of the human body (Figure 1). Increasing studies implicated that microbiota are critical to the development of chemoradiotherapy resistance, therefore a greater insight into microbiota is urgently required. The microbiota can interact with cancer treatments in a two‐way way; on the one hand, anticancer treatments can destroy the microbial composition in intestine and cause malnutrition in patients, then microbiota affects the effectiveness of cancer treatment on the other hand.^1^

**FIGURE 1:**
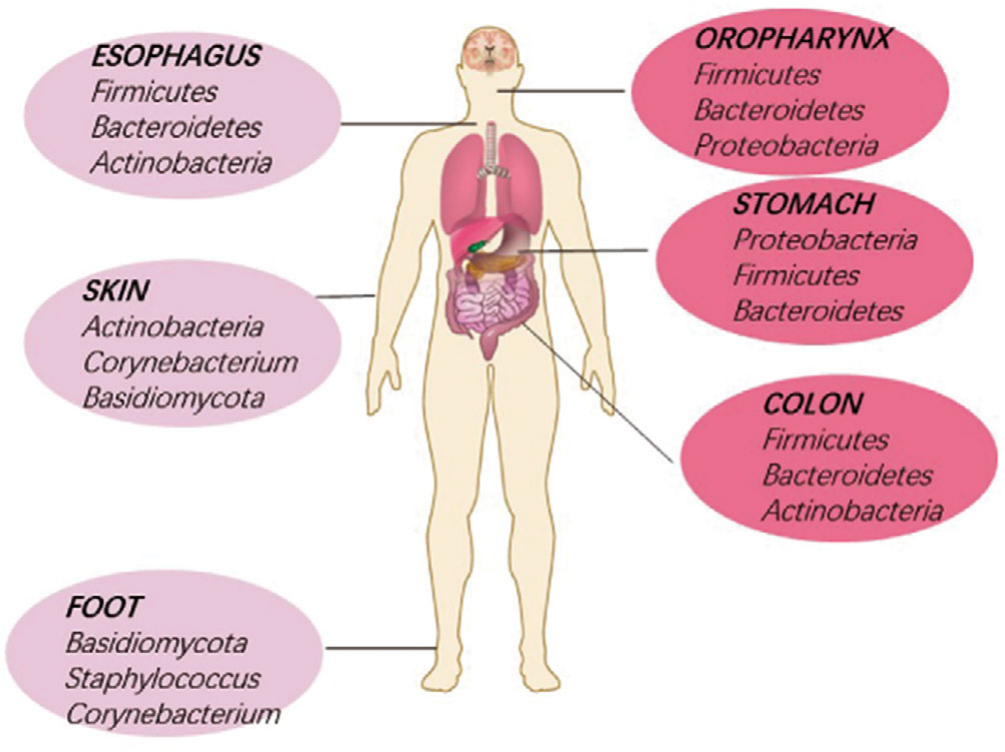
Microbiota in various tissues

Radiotherapy could break both single‐strand and double‐strand of DNA through radiation that penetrates tumor tissues directly and reduces tumor genotoxicity. The frequency and severity of the tumor radio sensitivity and the toxicity caused by radiotherapy is the main reason of resistance. Consequently, it is important for people to understand the correlations among the microbiota and radiotherapy sensitivity and toxicity. But research on the mechanism of radiotherapy resistance generation is not comprehensive, and the research is primarily focused on esophageal cancer, neuroglioma, lung cancer, and cervical cancer. For example, Cui et al found that alterations in the sorts and compositions of the gut microbiota affected the radiotherapy (RT) resistance in mice; data demonstrated that the decrease in the sorts of intestinal microbiota leads to a physiological disturbance in mice, and alterations in the composition of microbiota increase the resistance of mice to radiotherapy.^2^ Another study confirmed that the intestinal microbiota is critical in the efficiency of total body irradiation (TBI).^3^ Mice with tumor were treated with TBI, and then injected with pmel‐1 CD8^+^ T cells. However, when antibiotics were used before TBI, the abundance of gut microbiota decreased, which reduced the effectiveness of TBI and in turn reduced the effectiveness of the injected CD8^+^ T cells in cancer cells.^3^ Meanwhile, for the generation of radiation sensitivity and radiotherapy toxicity, microorganisms have been proved to be a potential key player. They may be involved in the regulation of immune responses^1^, ^4^ (Figure 2A and B). But further investigations are needed.

**FIGURE 2:**
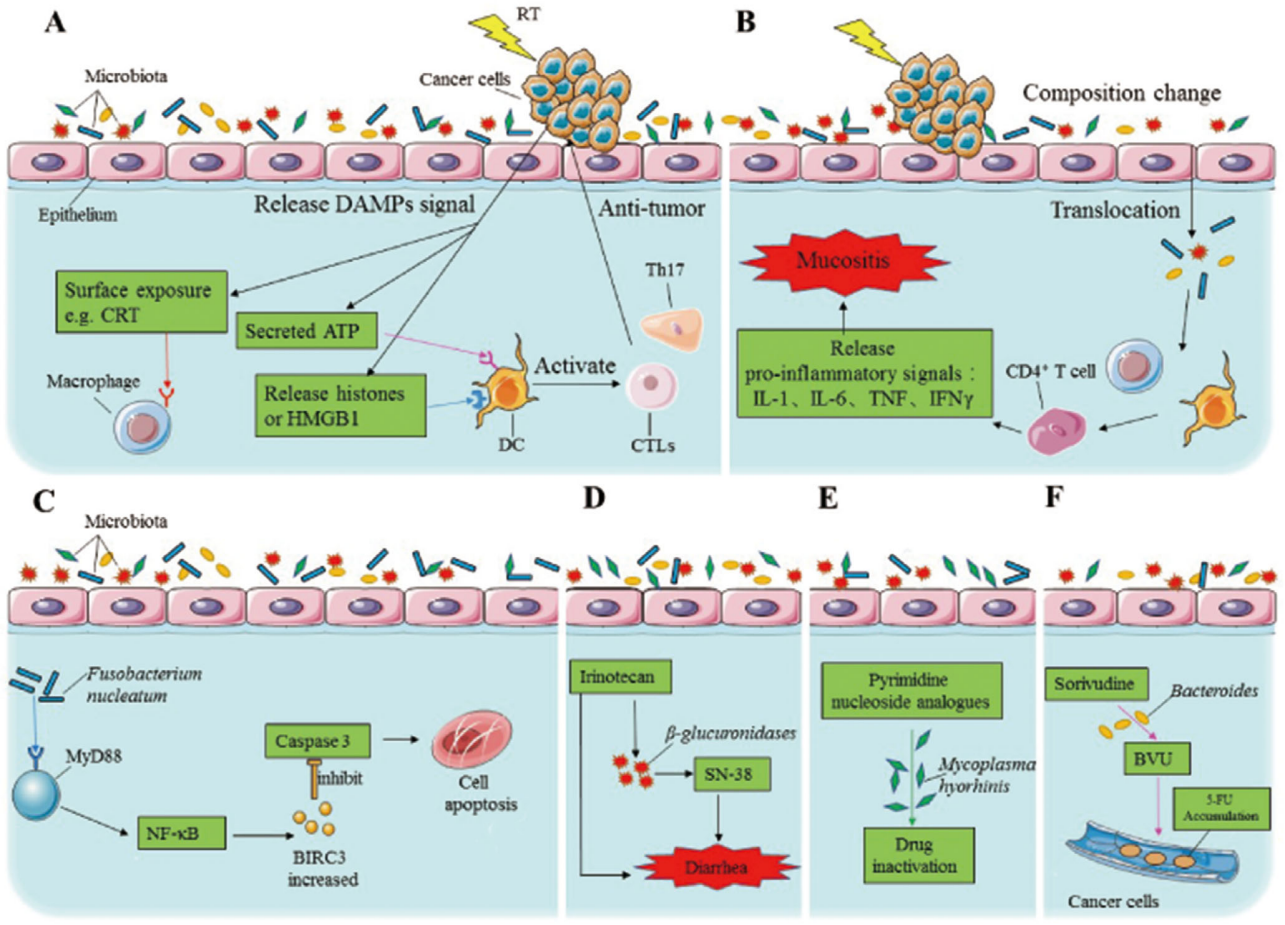
(**A**) RT induces local immunogenic effects. Radiation stimulates the generation of damage‐associated molecular patterns (DAMPs) in cancer cells, including CRT, extracellular ATP, histones, and HMGB1. These factors interact with immune cells like DCs and macrophages to further induce antitumor immune responses mediated by Th17 cells and CTLs. (**B)** The microbiota affects the antitumor response induced by RT. Radiation causes translocation of microbiota, while the components change. This disrupts the local immune balance and promotes the release of inflammatory cytokines, leading to mucositis. CRT, calreticulin; HMGB1, high mobility group box 1 protein; CTLs, cytotoxic T lymphocytes; Th17, T helper 17 cells; IL‐1, interleukin‐1; IL‐6, interleukin‐6; TNF, tumor necrosis factor; IFN‐γ, interferon‐γ. (**C)** Intratumoral bacterial *Fusobacterium nucleatum* could be involved in the 5‐FU chemoresistance by activating the innate immune pathway TLR4/MyD88. (**D)** Microbial *β‐glucuronidases* can give rise to an increase of the irinotecan active metabolite (SN‐38), which is associated with the occurrence of severe diarrhea. (**E)** *Mycoplasma hyorhinis* can directly degrade the pyrimidine nucleoside analogues (PNA) by its thymidine phosphorylase activity. (**F)** *Bacteroides*, dominant members of intestinal microbiota, have a high activity of sorivudine conversion to an intermediate (BVU), which inhibits the degradation of 5‐FU and results in its accumulation in the blood and then in a higher toxicity.

The relationship between chemotherapy treatment and the microbiota is more complex. One study verified the activation of autophagy through the immune pathway toll‐like receptor 4 (TLR4)/myeloid differentiation primary response protein 88 (MyD88) by *Fusobacterium nucleatum*, which is responsible for the chemoresistance to 5‐fluorouracil (5‐FU)^5^ (Figure 2C). Based on the past research, the gut microbiota probably causes a pharmacotherapeutics dilemma in the cancer therapy (Table S1). It has also previously been observed that the microbiota in the intestine can immediately metabolize xenobiotics, such as antitumor agents, leading to lower therapy efficacy. For example, one study pointed out that microbial β‐glucuronidases can activate the inactive form of irinotecan, and causes diarrhea^6^ (Figure 2D). Another study also showed that *Mycoplasma hyorhinis* can directly degrade the pyrimidine nucleoside analogues (PNA) by its thymidine phosphorylase activity^7^ (Figure 2E). And a newest study found that simply changing the amount of one amino acid in the diet (Serine) could completely alter the cellular and molecular mechanisms of fluorouracil and significantly affect its efficacy, which depend on the gut microbiota, rather than the subject of the drug.^8^ Besides its function in drug metabolism, the gut microbiota is involved in the toxicity of drug therapy in assorted tumors. Sometimes, this may lead to toxicity of the current chemotherapy agents. Sorivudine is a potent agent against virus, but it cannot be used in the combination with 5‐FU because a metabolite of sorivudine named BVU could inhibit the 5‐FU degradation and lead to the accumulation of 5‐FU in the blood and enhanced the toxicity^9^ (Figure 2F).

Thus, there should be a focus on potential strategies to reduce chemoradiotherapy resistance by targeting the gut microbiota. And it is also significant to concentrate on microbiota assessment in cancer patients. To safely target the microbiome, there are three methods that optimize the microbiota composition and improve the effectiveness of cancer treatment. Probiotics are generally considered as live microorganisms that have a positive effect on the host after ingestion. The use of probiotics can restore the natural balance of intestinal flora and the health status of the body. Fecal microbiota transplantation (FMT) is a new technology that directly changes gut microbiota of patients to normalize the composition and gain a therapeutic benefit. And antibiotics were first discovered as a class of basic metabolites with antipathogenic activity produced during microbial metabolism. Currently, antibiotics and FMT are the only FDA‐approved anticancer treatments targeting the gut microbiome^10^. They may alter the composition of the microorganism and reduce the negative impact of bacteria on anticancer treatment by adjusting the proportion of the microbiota.

In conclusion, these understandings on the role of the microbiota may provide solutions to solve cancer treatment side effects. However, evidence of the direct effects of the gut microbiota in different tumors is still urgently needed. Further studies are required to elucidate how the microbiota participates in cancer therapy and to identify novel approaches to improve cancer patient outcomes. Moreover, we need a methodology for exploring these host‐microbiota interactions in patients receiving chemoradiotherapy.

## CONFLICT OF INTEREST

The authors declare that they have no conflict of interest.

## FUNDING

National Natural Science Foundation of China; Project Numbers: 81672953 and 81803551; Guangdong Innovative and Entrepreneurial Research Team Program; Grant Number: 2013Y113; Zhuhai Innovative and Entrepreneurial Research Team Program; Grant Numbers: ZH01110405160015PWC and ZH01110405180040PWC; Guangzhou Science and Technology Project; Grant Number: 201904010061; The Fundamental Research Funds for the Central Universities; Grant Number: 21620429.

## Supporting information

Supporting information
