# Supplementary material for: Microbiota in cancer chemoradiotherapy resistance
Source: Clin Transl Med. 2020 Dec 31;11(1):e250. doi: 10.1002/ctm2.250 (PMC7774460; doi:10.1002/ctm2.250)
Supplement: Supplementary file 1 — Supporting information [file CTM2-11-e250-s001.docx]

**Table S1**. Summary of microbiota-related chemoresistance

| Microbiota or microbial intervention | Chemotherapy | Mechanism |  | PMID |  |
| --- | --- | --- | --- | --- | --- |
| *Gammaproteobacteria* | Gemcitabine | Microbiota can metabolize gemcitabine (2',2'-difluorodeoxycytidine) into its inactive form, 2',2'-difluorodeoxyuridine. |  | 28912244 |  |
| *Fusobacterium nucleatum* | 5-Fluorouracil | Upregulated BIRC3 expression via theTLR4/NF-κB pathway, reduces the 5-FU sensitivity in CRC. |  | 30630498 |  |
| *Fusobacterium nucleatum* | 5-Fluorouracil/oxaliplatin | Targeting of TLR4 and MYD88 and specific microRNAs to trigger the autophagy. |  | 28753429 |  |
| *Mycoplasma hyorhinis* | Gemcitabine | Mycoplasma-derived pyrimidine nucleoside phosphorylase (PyNP) activity indirectly potentiates deamination of gemcitabine. |  | 24668817 |  |
| Antibiotic treatment | Oxaliplatin/cisplatin | Reduces the production of reactive oxygen species and cytotoxicity after drug therapy. |  | 24264989 |  |
| Bacteroidetes | Ipilimumab | Generation of Bacterial-related B vitamin and deficiency of polyamine  transport linked to elevated risk  of colitis induced by CTLA‑4 blockade. |  | 26837003 |  |
| Firmicutes, Actinobacteria,  Proteobacteria | Carmustine, etoposide,  cytarabine and melphalan  combination | Reduced diversity and ecological network function. |  | 26147207 |  |
| Anaerobes, streptococci, *Bacteroides* | Methotrexate | Minimizes protection of enterocytes against harmful stimuli. |  | 25376667 |  |
